# Association tests based on the principal-component analysis

**DOI:** 10.1186/1753-6561-1-s1-s130

**Published:** 2007-12-18

**Authors:** Sohee Oh, Taesung Park

**Affiliations:** 1 Department of Statistics, Seoul National University, 56-1 Shillim-Dong, Kownak-Gu, Seoul 151-747, South Korea

## Abstract

Haplotypes are composed of specific combinations of alleles at the several loci on the same chromosome. Because haplotypes incorporate linkage disequilibrium (LD) information from multiple loci, haplotype-based association analyses can provide greater powers than the single-marker analysis in the association studies. However, when we construct haplotypes using many markers simultaneously, we may be confronted with a sparseness problem due to a large number of haplotypes. In this paper, we propose the principal-component (PC) association test as an alternative to the haplotype-based association test. We define the PC scores from the LD blocks and perform the association test using logistic regression. The proposed PC test was applied to the analysis of the Genetic Analysis Workshop 15 simulated data set. By knowing the answers of Problem 3, we evaluated the performance of the PC test and the haplotype-based association test using Akaike Information Criterion (AIC), power, and type I error. The PC test performed better than the haplotype-based association test in the sense that the former tends to have smaller AIC values and slightly greater power than the latter.

## Background

Recently, several studies have shown that the use of haplotypes may offer more powerful information on genetic association with traits than the use of single-nucleotide polymorphisms (SNPs) [1,2]. Haplotypes are specific combinations of allelic variants at a series of tightly linked markers on the same chromosome. Haplotypes incorporate linkage disequilibrium (LD) information from multiple loci. If markers have low LD relationship in the LD block, then a large number of haplotypes are constructed. Several methods have been proposed to test whether the haplotypes are associated with the disease trait. In these association studies, haplotypes are treated as covariates in logistic regression models [3-6]. However, the haplotype-based association test has a problem when the number of haplotypes is large. If there are *m *markers, then the maximum number of haplotypes is 2^*m*^. When there are many haplotypes, parameter estimation is difficult due to the large number of parameters as well as the sparseness of data.

In order to solve this problem, we propose the principal-component (PC) association test as an alternative to the haplotype-based association test. PC scores are derived from the LD blocks. The PC scores have the same amount of information as the haplotypes. In general, the first few PC scores tend to have the most of information about LD blocks. Thus, the use of the first few PC scores may produce the similar results to the use of full haplotypes with fewer parameters.

The proposed PC scores test was applied to the analysis of the Genetic Analysis Workshop 15 simulated data set (Problem 3), which includes 100 replicates. Each replicate contains a random sample of 1500 families with an affected sibling pair (ASP), and a randomly selected member of the offspring generation from each of the 2000 unaffected control families. By knowing the answers of Problem 3, we evaluated the performance of the PC test and the haplotype-based association test using Akaike Information Criterion (AIC) [7], power, and type I error.

## Methods

### Genotype data and sample

We used all 100 replicates from chromosome 6 sparse SNP data set. We first performed the transmission/disequilibrium test (TDT) [8] and Hardy-Weinberg equilibrium test for family data sets. We did not include the markers with minor allele frequencies < 0.01. We selected unrelated individual samples including one sib from each ASP family (1500 individuals) and 500 controls.

### LD blocks

We considered SNP markers with LD, D' > 0.7. We selected eight LD blocks, where LD Blocks 1 to 4 are known to be not associated with the RA and Blocks 5 to 8 are known to be associated with the RA. Each LD block contained two to six markers.

### Haplotype-based association test

For the selected LD blocks, their haplotypes and frequencies were estimated by the expectation-maximization (EM) algorithm. We then performed the haplotype-association tests by fitting logistic regression. In this association study, we pooled the minor haplotypes that have frequencies less than 0.05. The effect of haplotype can be assumed to be additive, dominant, or recessive. In our analysis, we assumed the additive effect of haplotypes and performed the test using haplo.glm [5].

### PC score association test

We first determined whether the effect of a SNP in LD blocks is additive, dominant, or recessive. If the effect of the SNP is additive, the SNP is coded as 0, 1, and 2 according to the number of minor alleles. On the other hand, for the dominant or recessive effect, it is coded as 0 or 1. Then, we performed the PC analysis with LD blocks and calculated the PC scores. For the given LD block, suppose there are *k *SNPs denoted by *s*_1_, *s*_2_,..., *s*_*k*_, where *s*_*k *_is coded as 0, 1, or 2. Then, the PC scores are defined as follows:

*PC*_*i *_= *e*_*i*_*'S*,

where *PC*_*i *_is the *i*^th ^PC score, *e*_*i *_is its eigenvector, and *S *= [*s*_1_, *s*_2_,..., *s*_*k*_] is the score vector of SNPs. In our analysis, we only assumed the additive effect of SNPs. We determined the number of PC scores in each block to account for 70% of total variation, which ranged from one to three. For these PC scores, we fitted logistic regression with PC score as covariates.

### Comparison of PC score and haplotype-based association tests

The association tests were performed using logistic regression with PC scores and haplotypes as covariates. Using Akaike Information Criterion (AIC), power, and type I error, we evaluated the performances of the PC test and the haplotype-based association test.

## Results

### AIC of PC score and haplotype-based association tests

We selected randomly one replicate (Replicate 48). For this replicate, Table 1 summarizes the LD blocks, the number of SNPs, the number of PC scores, and the number of haplotypes. Blocks 1 to 4 are not associated with RA, while Blocks 5 to 8 are.

**Table 1:** The results of the PC score based and haplotype-based tests using Replicate 48

					Association test via logistic regression^c ^*H*_0_: Global *β *= 0		AIC	
						
	LD block (SNP)	No. SNPs	No. PC score^a^	No. haplotype^b^	PC score	Haplotype	PC score	Haplotype
Blocks not associated with RA	1 (47~51)	5	1	3 (3)	0.4602	0.9947	**2252.880**^d^	2254.346
	2 (79~82)	4	1	3 (8)	1.4472	3.3423	**2251.893**	2254.004
	3 (356~361)	6	3	5 (16)	0.8239	3.3700	**2256.517**	2257.973
	4 (387~390)	4	2	4 (9)	2.7136	3.3676	**2252.627**	2256.012
								
Blocks associated with RA	5 (128~130)	3	1	3 (6)	15.0769	18.1052	**2238.264**	2239.058
	6 (140~144)	5	3	4 (14)	17.4702	5.5822	**2239.870**	2253.928
	7 (149~151)	3	2	3 (5)	10.1205	11.6309	**2245.220**	2245.644
	8 (162~163)	2	2	2 (4)	40.4704	40.3278	**2214.870**	2215.014

^a^We consider the haplotypes that have frequencies greater than 0.05, and PC score whose sum of variations explains more than 70% of the total variations.

^b^Number in parentheses is the number of estimated haplotypes. Haplotypes with frequencies less than 0.05 were pooled together and included in the model as one group.

^c^We test all *β *= 0 using the likelihood ratio test.

^d^Bold indicates the smallest AIC value.

Using the LR statistic we tested the global hypothesis that all *β *= 0. The LR statistics of Table 1 show that Blocks 1 to 4 are not significantly associated with RA and Blocks 5 to 8 are significantly associated with RA in both PC score and haplotype-based methods except for block 6. In addition, the degrees of freedom of PC score tests are smaller than those of the haplotype-based association tests.

Table 1 also shows that the association tests based on the PC scores and haplotypes have almost the same AIC values. However, the AIC values of the PC score tests are slightly less than those of the haplotype-based association tests. In addition, the degrees of freedom of PC score tests are smaller than those of the haplotype-based association tests.

**Figure 1:**
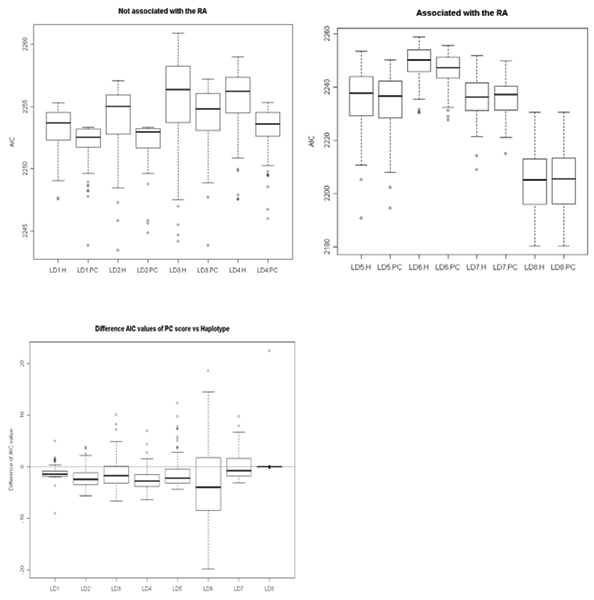
**Box plot of AIC from the all 100 replicates**. H, haplotype association test; PC, PC test.

Figure 1 shows the box plots of AIC values from all 100 replicates. The first panel shows the results of Blocks 1 to 4, which are not associated with RA. The second figure panel shows the results of Blocks 5 to 8, which are associated with RA. The y-axis is the value of AIC and the x-axis indicates whether the test is haplotype-based or PC score based. For example, LD1.H represents the result of the haplotype-based association test using LD Block 1 and LD1.PC does the result of the PC score test using the same block. The third panel shows the distribution of the differences of AIC values between the haplotype-based and PC score tests. In summary, the PC score test tends to have smaller AIC values than the haplotype-based test.

### Type I error and power

Using all 100 replicates, we performed the association tests based on the PC scores and haplotypes in order to compare the type I errors and powers. The type I errors were computed from the LD Blocks 1 to 4 and the powers were computed from the LD Blocks 5 to 8. Type I errors and powers were computed as the number of significant tests divided by the total number of replicates 100. Table 2 summarizes the type I errors and powers, showing that both tests preserved type I errors and the PC score test has greater power than the haplotype-based test.

**Table 2:** Type I errors and powers of PC score based and haplotype-based tests from the 100 replicates

	LD block		PC score	Haplotype
Type I error	Blocks not associated with RA	1	0.05	0.03
		2	0.03	0.04
		3	0.03	0.06
		4	0.03	0.03
				
Power	Blocks associated with RA	5	1.00	0.93
		6	0.78	0.42
		7	0.99	0.99
		8	1.00	1.00

## Discussion and conclusion

In this paper, we proposed using PC scores for the association test as an alternative to the haplotype-based test. The use of PC scores has the effect of reducing the number of parameters in logistic regression. The proposed method would be very useful when the number of haplotypes is large. In our analysis, the PC score test was shown to have smaller AIC values than the haplotype-based test, while the PC score test has a much smaller number of parameters.

PC analysis has been mainly applied to the analysis of quantitative variables. However, it has been successfully used to analyze the discrete SNP data mainly focusing on selection of SNPs. For example, Horne and Camp [9] proposed the PCA method for identification of LD groups and selection of optimal SNP-sets that capture sufficient intragenic genetic diversity. Lin and Altman [10] proposed using the PCA method to find haplotype tagging SNPs. Unlike these previous methods, our method focussed on association studies using PCA.

One drawback of the PC score test is that the interpretation of scores is not straightforward. In particular, the biological meaning of PC scores cannot be easily obtained. In our study, a significant result of PC scores implies that some SNPs in the LD block are associated with the disease. Among the SNPs in the LD block, the SNP which has the largest component of the eigenvector has the greatest impact on the disease.

The PC score test has many advantages. First, it has the effect of dimensional reduction. It reduces the number of parameters greatly. As a result, it can avoid the sparseness of data. Second, it can easily handle more complicated association studies such as gene × gene interactions. On the other hand, the haplotype-based test cannot easily handle gene × gene interactions across different chromosomes. In order to handle gene × gene interactions between different chromosomes, the haplotype-based approach need to consider the haplotype × haplotype interactions, which requires a much larger number of parameters and cannot be handled easily.

In summary, the proposed PC score method may be applied to the classification analysis and other interaction studies such as for the gene × environment interactions. Furthermore, PC scores are summary measures of LD blocks. Thus, we recommend these measures to be used for a possible construction of gene regulatory networks, which we will investigate in the future.

## Competing interests

The author(s) declare that they have no competing interests.
